# Wandering liver and intestinal malrotation: first report

**DOI:** 10.1186/s40792-016-0205-y

**Published:** 2016-08-06

**Authors:** Alex Ordonez, David Nguyen, Stephanie Mlacker, Andrea Ordonez, Emanuele Lo Menzo, Samuel Szomstein, Raul Rosenthal

**Affiliations:** 1 The Bariatric and Metabolic Institute, Section of Minimally Invasive Surgery, Department of General and Vascular Surgery, Cleveland Clinic Florida, 2950 Cleveland Clinic Boulevard, Weston, FL 33331 USA; 2 Department of General Surgery Residency Program and Fellowship in Minimally Invasive and Bariatric Surgery, Cleveland Clinic Florida, 2950 Cleveland Clinic Blvd, Weston, FL 33331 USA

**Keywords:** Wandering liver, Intestinal malrotation, Laparoscopic surgery, Hepatic suspensory apparatus

## Abstract

A wandering liver is a rare development in both the adult and pediatric population where the liver is freely displaced along a transverse axis. We describe the first known occurrence in published literature of a wandering liver in an adult individual who also had an intestinal malrotation complicated by a midgut volvulus. The abnormal ability for a liver to wander presents a highly unusual anatomy that can be disorienting. Laparoscopic surgery is a viable option in reducing a midgut volvulus and addressing an intestinal malrotation in the presence of a wandering liver. This unusual presentation educates clinicians to avoid potential misdiagnosis given the abnormal location of the duodenum, appendix, liver, and gallbladder.

## Background

*Wandering liver* is a term used to describe a rare development, resulting from a structural malformation in the hepatic suspensory apparatus, in which the liver is free to displace along the transverse axis around the inferior vena cava. Both congenital and—more commonly—acquired forms of wandering liver have been described [1]. This anomaly is thought to arise in conjunction with a persistent ventral mesentery, which may incite a volvulus of the stomach and bowel [2]. In this report, we describe the first known presentation of a wandering liver and intestinal malrotation in an adult resulting in acute midgut volvulus.

## Case presentation

**Fig. 1:**
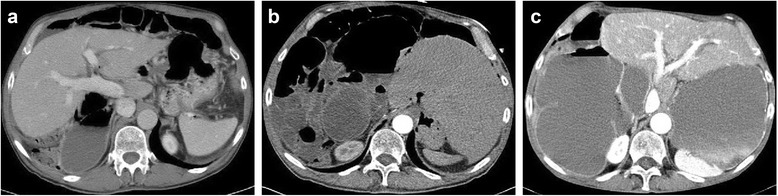
Transverse CT scans demonstrating the liver to be located to the left and right of the midline on different occasions. **a** Year 2009. **b** Year 2012. **c** Year 2014

**Fig. 2:**
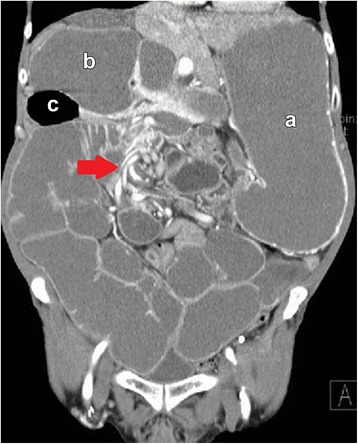
Coronal CT. Mesenteric volvulus illustrating a twisted configuration in the right abdomen with marked small bowel dilatation (*arrow*). Marked dilatation of the stomach (**a**); duodenum (**b**); and diverticulum (**c**)

A 69-year-old male presented with a long-standing history of nausea, vomiting, abdominal pain, and weight loss. Prior to this presentation, he was under the care of multiple gastrointestinal physicians for diet intolerance, bloating, and intermittent abdominal pain, which was attributed to diverticulosis of the small bowel. History also included intestinal malrotation and a wandering liver diagnosed from computed tomography (CT) scans. The liver on CT scans was demonstrated to be located to the left and right of the midline on different occasions (Fig. 1a–c). A diagnostic laparoscopy for presumed free intraperitoneal air 6 years prior also reported the presence of the liver on the left side. The intestinal malrotation was not addressed surgically at that point in time. The patient was transferred to our institution with a diagnosis of small bowel obstruction (SBO). An abdominal CT scan revealed distension of the stomach and small bowel with decompression of the mid-distal ileum and colon as well as a mesenteric volvulus. (Figure 2) The liver and gallbladder were located in the left upper quadrant and medially displaced relative to its position (to the right) noted from a prior CT scan performed 2 years earlier.

**Fig. 3:**
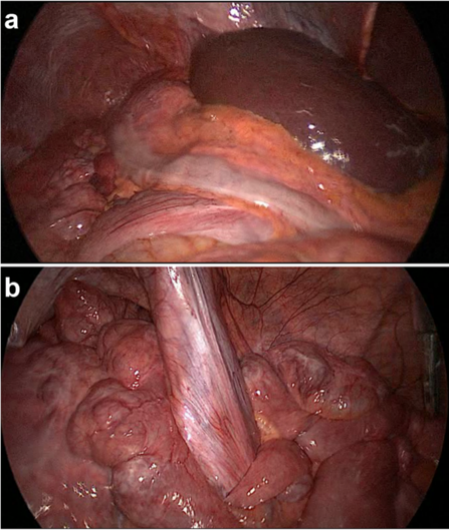
**a** Initial laparoscopic inspection revealing the liver to be located in the left upper quadrant in direct contact with the left abdominal wall; **b** Ladd’s band in the lower abdomen with surrounding diverticulosis of the small bowel

A decision was made to proceed with a diagnostic laparoscopy on the basis of a midgut volvulus, intestinal malrotation, and wandering liver on radiologic studies. Initial inspection revealed the liver and gallbladder to be located in the left upper quadrant in direct contact with the left abdominal wall. (Fig. 3a) Neither organ showed any obvious pathology. The stomach was found to be mildly dilated and located below the liver, with the pylorus oriented to the right upper quadrant. Dilatation of the proximal small bowel and multiple large diverticula arising from the duodenum was also evident. The transverse colon was located in the right upper quadrant, with the descending colon traversing down to the suprapubic area. The duodenum was also visualized in the right upper quadrant, containing numerous large diverticula without signs of active inflammation. The small bowel was run and rotated in a counterclockwise fashion. A volvulus of the mesentery was evident in the mid to distal small intestine, which was subsequently reduced. During counterclockwise rotation, the small bowel was placed in the right side of the abdomen with the large bowel moved to the left abdomen. A fibrotic tissue, corresponding to a Ladd’s band (Fig. 3b), was visualized crossing along a transverse plane in the lower abdomen and was taken down with ultrasonic scissors to the root of the mesenteric vessels. The small intestine was run again to verify the reduction of the volvulus and the absence of any twisting. The final procedure performed was an appendectomy.

Postoperatively, the patient experienced an ileus and recovered uneventfully while regaining bowel function and tolerating diet by postoperative day 3. At 2 weeks of follow-up, resolution of symptoms was demonstrated.

### Discussion

A wandering or hypermobile liver in both the adult and pediatric population is rare in nature and the reported literature. A total of 11 cases since 1960 have been published in medical journals. Various distinct pathologies are associated with this condition in volvulus and/or obstruction of the stomach and bowel due to a persistent ventral mesogastrium [2, 3]. To our knowledge, this is the first reported occurrence of a concurrent wandering liver and intestinal malrotation in an individual. The incident was additionally complicated by a midgut volvulus with diverticulosis in full length of the small bowel.

The hepatic suspensory apparatus, which suspends the liver from the diaphragm and maintains the liver in the right upper quadrant of the abdomen, is made up of the coronary, triangular, and falciform ligaments [3]. A persistent ventral mesentery is known to be associated with malrotation and volvulus; however, because the ligaments of the hepatic suspensory apparatus are derived from the ventral mesentery before it fuses with the peritoneum, it has been proposed that a persistent ventral mesentery could represent an arrest in the normal development of these ligaments, which predisposes them to laxity, allowing the liver to “wander” [2, 3].

Intestinal malrotation diagnosed at adult age is a rather rare finding and typically asymptomatic. Authors Nichols et al. [4] proposed an embryologic error that may result in the laxity or absence of both hepatic and colonic suspensory ligaments in the setting of colonic volvulus. We agree on this notion of developmental error; however, present literature does not offer any link of hepatic hypermobility to intestinal malrotation or volvulus. Nonetheless, malrotation is reported in 1:500 live births before one month of age with, essentially, no mention of a wandering liver [5]. The impression that a wandering liver is an independent entity as opposed to a clinical sign in a syndrome requires more reported incidences for epidemiologic investigation.

In adult life, intestinal malrotation is generally an incidental finding on CT [4] and exceptionally infrequent in the setting of a wandering liver. This combination can be discovered during abdominal surgeries for different reasons or present as bowel obstruction. Recurrent episodes of bowel obstruction can occur with the potential of partial or complete volvulus. When symptomatic, prompt intervention (which could be performed laparoscopically) is indicated. Thus, a systemic approach to intestinal malrotation with a wandering liver is crucial in recognizing abnormal anatomy (i.e., duodenal obstruction) especially when approached laparoscopically.

In this case presentation, the preoperative CT scan demonstrated the liver to be midline of anterior abdomen. Upon insufflation and entering the abdomen laparoscopically, the liver was positioned in the left upper quadrant. With manipulation of the liver and positioning of the patient on the operating table, the unimpeded laxity of the hepatic ligaments was manifested within the upper abdominal cavity. This may indicate the competency of the liver to wander in abbreviated times as opposed to a long migratory process. Although not established, the short wandering time may increase predisposition to bowel obstruction in the presence of intestinal malrotation. Figure 1 exhibits liver migration within years of each other. However, on basis of laparoscopic exploration, a wandering liver suggests the ability to migrate in varying locations within hours. Hepatic displacement appears to be independent of any intra-abdominal pathology, i.e., bowel obstructions.

In the treatment approach of concurrent intestinal malrotation and wandering liver, identifying Ladd’s bands is critical for long-term success. The Ladd procedure remains the initial and mainstay procedure for intestinal malrotation in reducing any volvulus, dividing mesenteric bands, and performing an appendectomy. Compared to the open approach, laparoscopic correction may result in a shorter hospital length of stay and incite fewer adhesions with improved outcomes in postoperative pain.

## Conclusions

This case explores the possibility for an association of both an intestinal malrotation and wandering liver in the adult population. An intestinal malrotation and midgut volvulus warrant prompt investigation and surgical intervention. It remains imperative that a wandering liver on incidental radiologic studies be monitored for the development of abdominal pathologies as varying hepatic displacements may occur in short timeframes. Clinicians must possess a high level of suspicion with this rare disorder to avoid potential misdiagnosis or delay in treatment given the abnormal location of multiple organs including the duodenum, appendix, liver, and gallbladder.

## Consent for publication

Written informed consent was obtained from the patient for publication of this case report and any accompanying images. A copy of the written consent is available for review by the Editor-in-Chief of this journal.
